# Marijuana use: neuroscience perspective

**DOI:** 10.3325/cmj.2014.55.281

**Published:** 2014-06

**Authors:** Lukasz M. Konopka

**Affiliations:** Department of Psychiatry, Loyola Medical Center, Maywood Il, USA and Yellowbrick Consultation and Treatment Center, Evanston IL, USA

The human behavioral repertoire includes activities that influence mood and cognition such as listening to music, watching movies, conversation, and dining, behaviors considered within the normal spectrum; however, when we choose activities that predictably and reliably alter our mood and cognition by exposing our central nervous system to exogenous molecules, we enter the potentially pathological path of addiction. We, and others around us, begin to worry as these behaviors insidiously become dominant foci in our existence. We do not clearly understand why a given individual chooses to use recreational drugs (1). As a society, we interpret and judge substance use based on our cultural prejudices, current moral and legal standards, and established norms.

Today, with varying levels of acceptance throughout the world, the four, most abused, psychoactive substances are: caffeine, nicotine, alcohol, and cannabis. Often, when individuals abuse one of these substances, they eventually migrate to using all four. Cannabis recently has gained significant social acceptance. People believe cannabis is benign, has few significant detrimental brain effects, and is also non-addicting. These are false popular misconceptions (2). Although the human brain naturally produces and uses cannabis-like molecules, referred to as endocannabinoids, current scientific data show that marijuana’s externally administered substances directly influence behavior by affecting brain functions (3).

The human endogenous endocannabinoid system indicates that natural substances that contain cannabis-like molecules will interact with the nervous system through existing receptors. Cannabinoid receptors are present in the peripheral cells such as the immune cells and neurons of the central nervous system including the spinal cord, cerebellum, basal ganglia, periaqueductal gray, hippocampus, and neocortex (4). In the central nervous system, these neuronal receptors play a significant role in modulating behaviors and brain functions. Data suggest that cannabinoid receptors play a more direct role in brain functions such as pain modulation, motor activity, reduced rapid eye-movement sleep, as well as mood, motivation, and higher cognitive processes (5). Their impact may combine direct and indirect cellular and network effects. The cannabinoid system’s neurotransmitter molecules are atypically synthesized in the cellular membrane by a complex enzymatic process of dendritic lipid metabolism (receptive and integrative structure of the neurons) (6). Although they behave similarly to classical neurotransmitter systems, unlike many other classical neurotransmitters, these neurotransmitter molecules are not stored in specialized vesicles. Current published data suggest that the endocannabinoid nervous system’s primary effect is to influence neurotransmitter release through presynaptic modulations. Endocannabinoid receptors have been characterized in two different receptor types, type-1 and type- 2 (7,8). Type-1 receptors are primarily found in the central nervous system, whereas type-2 receptors are thought to be primarily distributed to the peripheral system. Current data also show that type-1 receptors are closely associated with excitatory neurotransmitters. Based on how these receptors relatively distribute within a given system that contains inhibitory and excitatory neurons, type-1 receptor activation may cause either inhibition or excitation that may translate to physiologic seizure inhibition or facilitation, which is considered pathological neuronal activity (9,10). Receptor distribution may vary from person to person; therefore, based on cannabis use, we may expect individually defined, variable effects that eventually translate into unexpected behaviors.

Marijuana and marijuana extracts are not only used recreationally: cannabis has a long history of medical treatment of various medical and neurological illnesses. Although recently it has gained the attention of research, the biochemical analysis of marijuana’s bioactive extracts is moving forward; this may give researchers significant understanding of the endocannbinoid system and how to develop new therapeutic agents that target that system. Already, significant advances have been made in managing chronic pain, glaucoma, head injury inflammatory disorders, and nausea, to mention a few (11).

Despite marijuana’s tremendous medical potential, this drug’s recreational use presents medical and social problems (12-14). Recreational cannabis use has significantly increased throughout the world (15). As marijuana’s public acceptance increases, the age of marijuana exposure decreases. In the US, data indicate that while nicotine use has decreased among college students, cannabinoid use has increased. In the light of these findings, we must ask ourselves whether there are significant consequences for chronic cannabis use. We also need to ask about the frequency of cannabis use and its direct impact on brain function. In recent years, as marijuana’s quality has improved, the higher cannabinoid concentration has resulted in significantly increased potency. Cannabinoid concentration has increased up to five times, which has increased its biological effect on the brain and, more significantly, endangered vulnerable individuals.

Emerging behavioral and imaging data argue against recreational marijuana use because they show a relationship between the age of first use and the intensity of measurable consequences. Individuals who routinely use marijuana have significantly altered brain structures and functions with significantly altered cognition and mood regulation. Relative to non users, chronic marijuana users generally underperform in memory and cognitive tasks; in addition, chronic marijuana users may develop significant mood deregulation that presents as depression, anxiety, and may result in long-lasting amotivational syndrome. Current data are unclear about the duration of these effects. More alarming data argue that some chronic marijuana users can develop psychosis that, in some cases, may lead to significant chronic psychiatric conditions such as schizophrenia spectrum disorders (16).

Who are these vulnerable individuals? Although we have limited data regarding this issue, some trends are emerging. Indeed, it appears that a subpopulation of individuals may be particularly vulnerable to marijuana use. In these individuals, certain brain molecules have altered genetic regulation of certain neurotransmitters that include dopamine (17). This makes these individuals more likely to develop psychosis and potentially more vulnerable to developing chronic mental illness because these brain molecules help eliminate dopamine after its release. In addition to genetic vulnerability, it appears there is a well-defined window linked to age: Individuals who engaged in heavy marijuana use before age 17 are five times more likely to abuse other substances later in life (18). Furthermore, these vulnerabilities indicate that marijuana use may lead to future cognitive difficulty such as lower IQ and psychiatric symptoms (19).

Many researchers would agree that pre-teen, teen, and young adult marijuana use may be significantly detrimental to brain development and, consequently, to successful participation in life. Emerging data show that chronic marijuana users performed poorly in school, often found themselves in conflict with the law, and did not progress as expected in developing their interests and careers. One proposed hypothesis states that marijuana use leads to decreased motivation and decreased ability to focus, pay attention, form memories, and solve complex problems. It is becoming clear that chronic marijuana use impacts emotions as well as cognition and affects structures such as the hippocampus, critical for memory formation and retrieval; the amygdala, critical for the emotional brain; and the frontal lobes, critical for the executive brain (20).

Lastly, but not surprisingly, recreational marijuana use and other substance use such as caffeine, nicotine, and alcohol may significantly impair the performance and socially beneficial activity of our youth. As the youth become young adults and attempt to manage new challenges and the responsibilities of independence, they often face significant periods of stress. Their successful transition through these periods requires cognitive and emotional stability. Without these resources, when they face challenges, young adults often seek easy immediate solutions. If substance use is in their historical behavioral repertoire, they turn to substances for relief and as a solution for their problems. These behaviors tend to be strongly reinforcing and often result in a viscous cycle of addiction. Unfortunately, we currently cannot predict who will withstand the challenges addiction, who will move-on unharmed, or who may be doomed to lifelong struggles and significant psychiatric sequels.

It appears we cannot prevent experimentation with addictive substances; however, we can invest in effective biological education about addiction and its potential consequences. We also need to have open, honest, evidence-based, public, conversations that address the individual risks of substance use and abuse.
